# Chronic starvation induces microglial cell depletion in an activity-based anorexia model

**DOI:** 10.1038/s41598-025-98237-z

**Published:** 2025-04-23

**Authors:** Valerie Verspohl, Miranda van Egmond, Lilly Kneisel, Friederike Reese, Anna C. Thelen, Nele Korten, Maren Neumann, Lena Schaack, Clara Voelz, Larissa Käver, Beate Herpertz-Dahlmann, Cordian Beyer, Jochen Seitz, Stefanie Trinh

**Affiliations:** 1 Institute of Neuroanatomy, RWTH Aachen University, Wendlingweg 2, 52074 Aachen, Germany; 2 Department of Child and Adolescent Psychiatry, Psychosomatics and Psychotherapy, RWTH Aachen University, Neuenhofer Weg 21, 52074 Aachen, Germany; 3 Department of Child and Adolescent Psychiatry, Psychosomatics and Psychotherapy, LVR-University Hospital, University of Duisburg-Essen, Wickenburgstraße 21, 45147 Essen, Germany

**Keywords:** Astrocyte, Microglia, Oligodendrocyte, Translational research, Gene expression analysis

## Abstract

Anorexia nervosa (AN) is a severe psychiatric disease with a largely unknown pathophysiology. AN leads to reduced brain volume and a disbalance of the gut microbiome suggesting the involvement of the gut-brain-axis. Also, in the activity-based anorexia (ABA) animal model mimicking AN brain volume loss is observed. This study investigated the impact of chronic starvation on brain cell populations and evaluated the potential protective effects of omega-3 fatty acids (FA) and probiotics in rats. We used a chronic ABA model and provided daily oral supplementation of omega-3 FA and probiotics. Immunohistochemistry and qPCR were used to analyze GFAP-positive astrocytes, IBA1-positive microglia, OLIG1/2-positive oligodendrocytes, MAP2-positive neurons and Ki-67-positive proliferating cells in the cerebral cortex and corpus callosum. We found a significant reduction of astrocytes and microglia in all ABA groups, likely due to reduced proliferating cells. Reduced running wheel activity and reduced amount of food needed to sustain body weight were observed in animals with supplementation with omega-3 FA and probiotics but we did not observe alterations in brain cells that could be attributed to these supplementations. Our results indicate that glial cell depletion potentially underlies the diminished brain volume found in ABA rats. Omega-3 FA and probiotics show potential for reducing AN-related symptoms and merit further study as a therapeutic approach.

**Supplementary Information:**

The online version contains supplementary material available at 10.1038/s41598-025-98237-z.

## Introduction

### Anorexia nervosa

Anorexia nervosa (AN) is a psychiatric disorder characterized by severe weight loss caused by insufficient energy intake, often in combination with increased physical activity^1^. AN has the highest mortality rate among psychiatric disorders and is one of the most common chronic diseases of adolescence^2,3^. It has drastic somatic consequences, including neurological, endocrine, and cardiovascular problems^4,5^. The prevalence of AN is increasing^6,7^, with a current lifetime prevalence of up to 4% in girls and 0.3% in boys^8^. The incidence of AN peaks in adolescence, with an onset of 50% by the age of 18^9^. The etiology is multifactorial and involves significant contributions from social, environmental (including the COVID-19 pandemic), and genetic factors^10^. Despite the multimodal treatment approach^11^, many patients experience relapses or chronic disease progression^12^.

### ABA model

To mimic acute AN in rodents, the classical activity-based anorexia (ABA) paradigm based on self-starvation (restricted feeding and unlimited running wheel access) was developed^13^. It is the most commonly used model to induce physical, hormonal and behavioral characteristics of AN including rapid weight loss and excessive physical activity^14^. Later, a chronic version of the model was developed representing the chronic course of AN by maintaining reduced bodyweight, additionally reducing the high mortality rate of the classical ABA model^15^. The chronic ABA model therefore reflects AN as a chronic disease by capturing long-term effects and enables the implementation of interventions.

### Brain volume and cell populations in response to ABA

One of the most striking consequences of AN is the reduced brain volume observed in patients. There is a loss of 7.6% of gray matter (GM) and 3.2% of white matter (WM) in adolescents^16–18^. Short-term weight rehabilitation increases both GM and WM volume^19^. Complete normalization remains elusive even after long-term weight rehabilitation^16,20–22^. Chronic weight loss due to undernutrition with long-term volume reduction is perceived to lead to long-term functional brain alterations and neuropsychological deficits^16,23^.

### Microbiome und supplementation

The microbiome refers to the genome of all microorganisms^24^. Although the majority of prevalent microbial genes are known, there is no definition of a standard healthy microbiome^25^. The individual composition is influenced by dietary and lifestyle habits^26,27^. Changes in the microbiome composition are observed in patients with AN^28^ and in the ABA model^29,30^, with partial normalization upon refeeding in both humans and rodents^31–33^.

Supplementation with omega-3 fatty acids (FAs) reduced gut permeability by influencing the microbiota composition and reducing inflammation through the production of anti-inflammatory products^34^. Omega-3 FAs administration improved cognitive function in young patients (7–14 years) with mood disorders^35^. Similarly, research showed that probiotic interventions had anxiolytic and antidepressant effects in patients with depressive symptoms^36^. In addition to their beneficial effects on other psychiatric diseases, omega-3 FAs and probiotics could be used to influence the microbiome composition in patients with AN and thereby improve AN-specific symptoms by affecting the gut-brain axis^35,37,38^.

### Hypothesis

This study aimed to examine the impact of chronic starvation on glial and neuronal cell populations. We hypothesized that, consistent with previous reports, there would be a reduction in astrocytes and alterations in other glia cells such as microglia, while neuronal cell counts were anticipated to remain unaltered^39,40^. We further explored the potential protective effects of supplementation with omega-3 FAs or probiotics on ABA animals by targeting the gut-brain axis. For this purpose, we specifically investigated cell count reductions and behavioral parameters of nutritional intake, physical activity, and weight loss. These results should broaden our understanding of the complex neurological mechanisms involved in AN and help uncover potential new therapeutic approaches.

## Materials and methods

### Animal experiments

We included 59 female, 4-week-old Wistar rats (Janvier, France), which were single-housed with a built-in running wheel connected to a tachometer (Sigma BC 5.12). Experimental conditions included room temperature at 23 °C, 55% humidity, and a 12-hour light/dark cycle (lights on at 7:00 am). Our sample size was determined based on the expected weight gain differences between the control and treated groups. We used a variance analytical model focusing on weight changes as the outcome. Pairwise comparisons were conducted using linear contrasts at an adjusted significance level of α = 5% (two-sided). Sample size estimation was based on a two-sided Satterthwaite t-test for unequal variances. Assuming a low dropout rate (maximum 5%), the estimated sample size was 15 per group. This calculation ensured statistical power with a type I error probability of 5% (global, two-sided) and a type II error probability of no more than 20%, aiming for at least 80% power. The animal facility was specific pathogen-free according to the FELASA guidelines and was certified according to DIN ISO 9001:2015. The North Rhine-Westphalia State Office for the Environment, Nature, and Consumer Protection (LANUV) approved all animal experiments for this study. All experiments were in compliance with the German legislation on animal experiments (National Research Council Committee for the Update of the Guide for the Care and Use of Laboratory Animals, 2011) and were conducted in accordance with directive 2010/63/EU concerning the protection of animals used for scientific purposes (European Parliament, 2010) and in accordance with the “Animal Research: Reporting of In Vivo Experiments” (ARRIVE) guidelines^41^.

### Study design

**Fig. 1:**
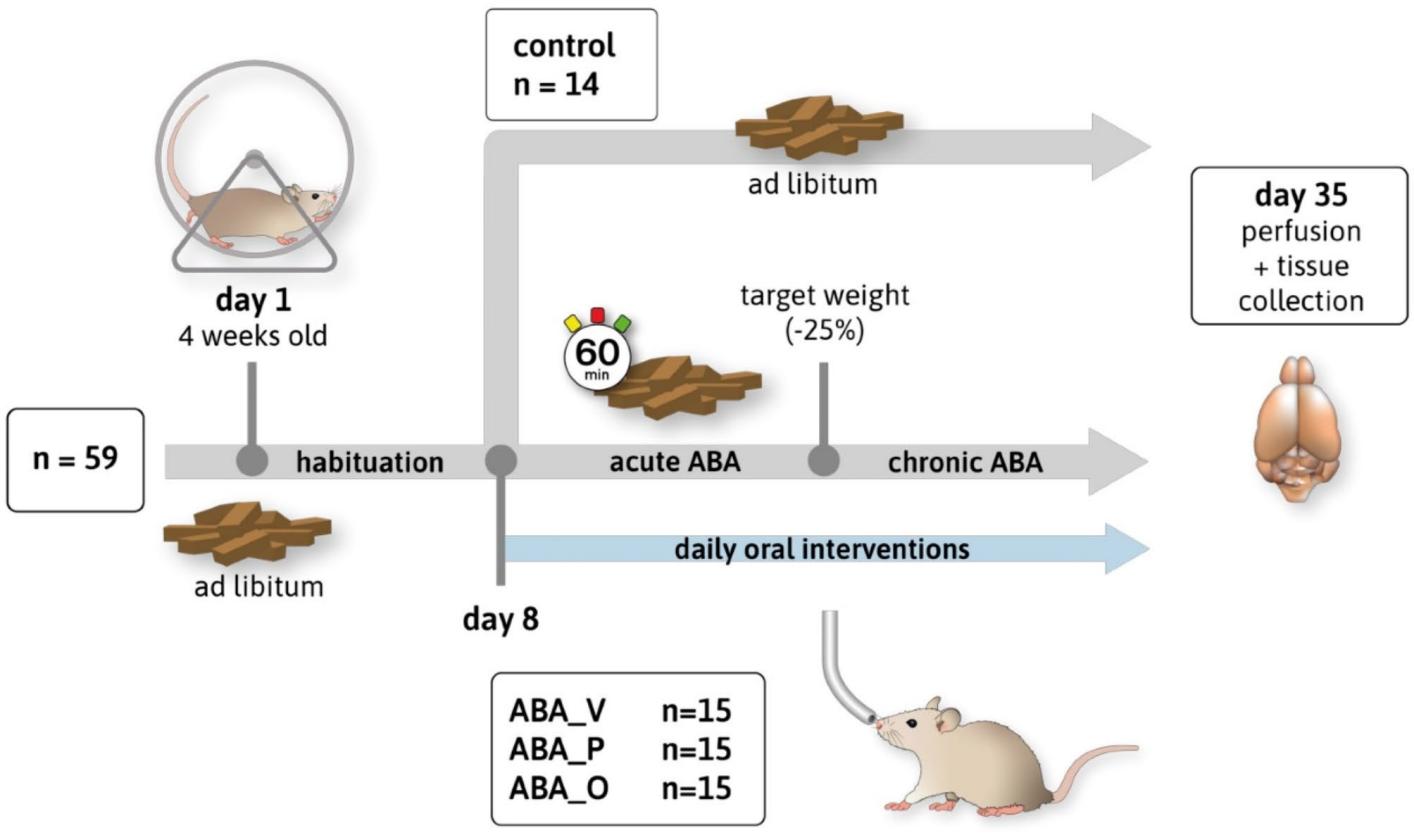
(**a**) A schematic summary of chronic starvation in 4-week-old rats. Daily measurements of body weight, running wheel activity and food intake were obtained. The acute and chronic starvation phases varied in length because rats reached the target weight after individual numbers of days.

Rats were randomly assigned to one of four groups (Fig. 1): the control group (*n = 14*), the vehicle intervention group (ABA_V, *n* = 15), the omega-3 FAs intervention group (ABA_O, *n* = 15), or the probiotic intervention group (ABA_P, *n* = 15). One control animal died before the start of the experiment. ABA-treated animals received daily oral interventions: ABA_V = 1 ml of water, ABA_O = omega-3 FAs from Opti3 Vegetology^®^ (volume in ml equaled 1% of body weight), ABA_P = 40 mg of OMNi-BiOTiC^®^ SR-9 dissolved in 1 ml of H_2_O^42^.

In the present study, the chronic version of the ABA paradigm was used. After a one-week acclimatization period with unlimited access to a running wheel and ad libitum food, the habituation phase began (days 1–7) to accustom the rats to daily handling. Handling data, such as body weight, running wheel activity (RWA) and food intake, were recorded daily (at 12 p.m.) for each rat throughout the experimental period. Control animals had unlimited access to a running wheel and food. On day 8, the acute ABA starvation protocol and oral interventions started. Specifically, ABA-treated animals were exposed to a limited feeding time of 1 h per day (1–2 p.m.)^13^ to allow detailed examination of eating behavior (food intake). An averaged offering of 100 µl omega-3 FA contained 1.73 kcl, while an average offering of 40 mg probiotics contained a negligible amount of calories (0.13 kcl). The used rat chow contains 3.32 kcl/g, so ABA_V and ABA_P animals received approximately 0.5 g extra food in the acute starvation period to compensate additional caloric intake due to omega-3 FA. When rats reached their individual target weight (defined as -25% of body weight on the last day of habituation), the chronic starvation phase started. In the chronic phase, the amount of food was individually adjusted as established by Frintrop et al.^15^, to maintain the specific target weight, so no additional food supply for ABA_V and ABA_P was needed to compensate for the omega-3 FAs. For statistical analysis, 0.5 g/day were deducted from the food intake data for ABA_V and ABA_P in the chronic starvation phase. The chronic starvation phase had an individual duration of 13–25 days, depending on the duration of reaching the target weight. At the end of the experiment, after 35 days, the rats were sacrificed by administering an overdose of isoflurane (Piramal, Mumbai, India). Subsequently, transcardial perfusion with 150 ml of phosphate-buffered saline (Thermo Fisher Scientific, Waltham, USA) was performed, and the cerebral cortex (CX) and corpus callosum (CC) were collected.

### Immunohistochemistry

For immunohistochemistry (IHC), 5 μm thin paraffin slices (frontal cutting, bregma − 2.30) were cut with an RM2255 microtome (Leica, Wetzlar, Germany). IHC was performed on two sections per animal according to a standard protocol^43^. For antigen retrieval, brain sections were first deparaffinized with xylol and a descending alcohol series, followed by heat-induced unmasking using either citrate or Tris-EDTA buffer. The slides were blocked with normal horse or normal goat serum diluted in PBS and diluted primary antibodies as listed in Table 1 were applied. After blocking with 0.3% H_2_O_2,_ the appropriate secondary antibody was applied followed by the Avidin/HRP-Biotin Coupling (VECTASTAIN Elite ABC Kit Standard; Vector Laboratories, California). After applying diaminobenzidine, the slides were counterstained with Mayer’s Hematoxylin. Then, slides were dehydrated in an ascending alcohol series and xylol. After embedding, slides were stored at room temperature.

**Table 1:** Antibodies used for immunohistochemistry.

Antibody	Antibody host	Concentration	Antigen-retrieval	Manufacturer	Target cells	Catalog number
GFAP	Mouse	1:2000	Tris-EDTA buffer (10 min)	Santa Cruz, USA	Astroglia	sc-33,673
IBA1	Rabbit	1:10000	Tris-EDTA buffer (10 min)	Wako, Germany	Microglia	019-19741
OLIG2	Mouse	1:1000	Tris-EDTA buffer (20 min)	Millipore, Germany	Mature and premature oligodendrocytes	MABN50
MAP2	Rrabbit	1:1000	Citrate buffer (20 min)	cell signaling, Netherlands	Neurons	8707
Ki-67	Rabbit	1:1500	Citrate buffer (10 min)	Abcam, UK	Proliferating cells	ab16667
APC	Mouse	1:250	Tris-EDTA buffer (20 min)	Millipore, Germany	Mature oligodendrocytes	OP80

### Quantification of positive cells

**Fig. 2:**
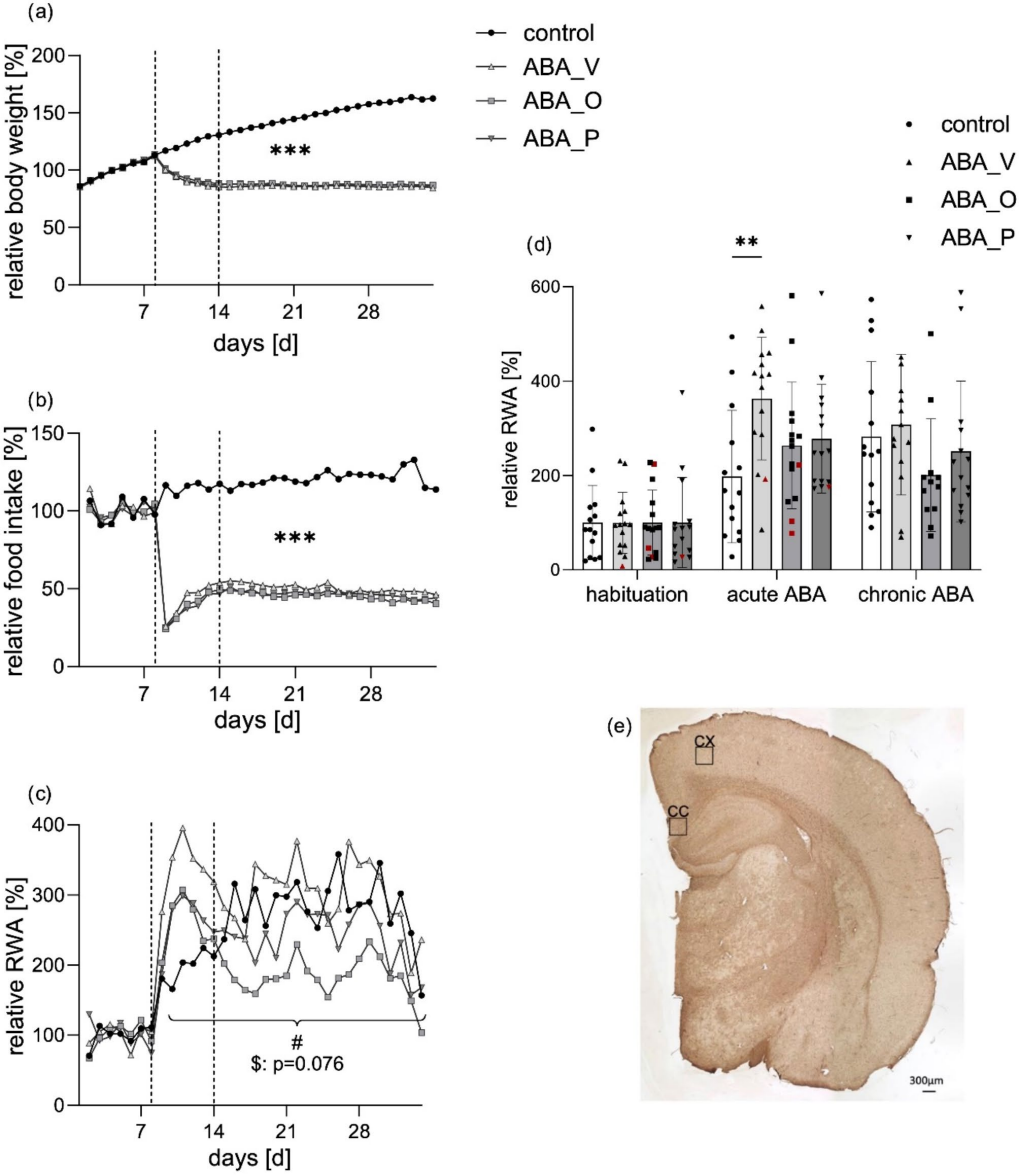
(**a**) Mean body weight per day normalized to body weight at habituation (days 1–7). ***p ≤ 0.001, repeated-measures ANOVA (rmANOVA) with post hoc Bonferroni correction over the complete starvation phase between control and all ABA animals (**b**) Mean food intake per day normalized to food intake at habituation (days 1–7). ***p ≤ 0.001, rmANOVA with post hoc Bonferroni correction over the complete starvation phase between the control and all ABA-treated animals. (**c**) The mean running wheel activity (RWA) normalized to the RWA at habituation (days 1–7), rmANOVA with post hoc Bonferroni correction over the complete starvation phase. #: significant changes (*p* = 0.018) for rmANOVA between ABA_O and ABA_V, including daily individual values; $: *p* = 0.076 indicates a trend toward a lower RWA in ABA_P than in ABA_V. (**d**) Mean running wheel activity (RWA) per phase normalized to RWA at habituation (mean ± SD), *n* = 59 (control = 14, ABA_V = 15, ABA_O = 15, ABA_P = 15), **p ≤ 0.01, two-way ANOVA with post hoc Bonferroni correction. Individual values from resistant animals are highlighted in red. (**e**) Representative image (half brain) of GFAP staining from a control animal. Enlarged images for evaluation were taken in regions of interest: the cortex cerebri (CX) and corpus callosum (CC). Control group with food ad libitum and no interventions, *ABA_V* ABA animals with water interventions, ABA_P ABA animals with probiotic interventions, *ABA_O* ABA animals with omega-3 FAs interventions.

The visualization of positive cells with visible nucleus was performed in predefined regions of interest (ROIs): the CX was chosen as an example of GM and the CC as an example of WM. Staining for the following antibodies was performed: Glial fibrillary acidic protein (GFAP), ionized calcium-binding adapter molecule 1 (IBA1), oligodendrocyte transcription factor 2 (OLIG2), adenomatous polyposis coli (APC) and the marker of proliferation Kiel 67 (Ki-67) Analysis of microtubule-associated protein 2 (MAP2) was performed in CX (GM). CC was analyzed at the midline, CX was analyzed in the center between CC and the top edge of CX (Fig. 2e). Digitization was achieved with a Nikon Eclipse 80i (Nikon, Tokyo, Japan). Ki-67-positive slices were imaged with a Keyence BZ-9000 microscope (Keyence, Osaka, Japan). Positive cells (with visible nuclei) were manually counted by two blinded independent observers with ImageJ software (1.52a, USA). The results were averaged and are displayed as cells/mm^2^.

### Reverse transcription (RT) and semiquantitative real-time polymerase chain reaction (qPCR)

The mRNA from the CX and CC was isolated using RNA-Solv (Omega Bio-Tek, Norcross, USA). Subsequently, these samples underwent reverse transcription into cDNA using an M-MLV RT-kit and random primers (Thermo Fisher Scientific, Waltham, USA). To verify specificity of the PCR amplification, an agarose gel was used to check the size of the PCR product. We used *cyclophilin A* as a reference gene, assessing constant expression by graphical inspection. Relative mRNA expression was quantified by calculating the ratio between the gene of interest (Table 2) and the reference gene (*cyclophilin A*). PCR runs were evaluated with the Δ Δ-cq method after quality control in the CFX Manager of the Bio-Rad software (version 3.1, Feldkirchen, Germany). The quality control included runs with an efficiency of 90–110% and excluded negative template controls with a Cq value less than 38, positive controls with a Cq value greater then 30, and replicates that differ with a standard deviation of the Cq value greater than 0.5. Changes in the gene levels of interest (Table 2) are presented as the fold change relative to the control groups, with the controls set to 100%.

**Table 2:** Primers for real-time polymerase chain reaction, *s* sense; *as* anti-sense.

Primer	Annealing temperature	Direction	Nucleotide sequence
*cyclophilin A*	65 °C	s	5′-GGCAAATGCTGGACCAAACAC
		as	5′-TTAGAGTTGTCCACAGTCGGAGATG
*GFAP*	61 °C	s	5′-AGAAAACCGCATCACCATTC
		as	5′-GCACACCTCACATCACATCC
*AIF1*	65 °C	s	5′-TGGAGTTTGATCTGAATGGCAATG
		as	5′-AGCCACTGGACACCTCTCTA
*OLIG1*	63 °C	s	5′-CGAGCGGAAGCGCAGCAGGA
		as	5′-AGAGCGAACTGGCCGCACG
*MAP2*	58 °C	s	5′-GCAAAGTAAGCCTGGTGA
		as	5′-ATCTAAGGGAAGAGTGAAAC
*Ki-67*	64 °C	s	5′-CTGCAGAGAAGGTTGGGATAAA
		as	5′-CTGACTTTGCCCAGAGATGAA
*APC*	65 °C	s	5′-ATAGCCTGCCCTTGACTGAG
		as	5′-TCTGCCGTGCTTCATACTCC

### Statistical analysis

The data of body weight, food intake and RWA are shown as the mean per day for each group and were normalized to the values of the habituation phase of each animal individually. Data of days until target weight, relative RWA per phase and weight at the start of the experiment are expressed as mean with the corresponding standard deviation (SD). IHC data are shown as the mean cell count with SD. qPCR data are shown as relative gene expression with the corresponding SD. GraphPad Prism (9.4.1., La Jolla, USA) and IBM SPSS statistics (29.0.0.0, Armonk, USA) were used for statistical analysis and visualization. The normality of the distribution was tested using either the Shapiro-Wilk test or Spearman’s rank test. If one test responded significantly, a box-cox transformation with consecutive analysis of variance (ANOVA) was performed to normalize the data. We performed one-way ANOVA to test for differences in the time to reach the target weight, for the IHC data and for qPCR data. To handle data that included daily individual values, we performed repeated-measure ANOVAs (rmANOVAs) separately for acute starvation (day 8-target weight) and total starvation episodes (days 8–35) on food intake, body weight and RWA. A two-way ANOVA was used to compare relative RWA in different starvation phases. After ANOVA calculation, a subsequent Bonferroni post hoc correction was performed to compare group differences. Statistical significance was set to 5%, and the trend-level set to 10%. Significant outliers were identified using the Grubbs test^44^. Individual values from resistant animals are highlighted in red in the figures.

## Results

### Starvation is sufficient to maintain reduced body weight and increased running wheel activity

At the beginning of the experiment, the average weight of the 4-week-old rats was 108.54 ± 9.51 g. The ABA-treated animals reached their target weight after a mean of 6.58 ± 2.93 days of acute ABA treatment (ABA_V = 5.78 ± 2.63, *n* = 14; ABA_O = 6.83 ± 3.48, *n* = 12; ABA_P = 7.14 ± 2.71, *n* = 14, Table 3, Supplementary Fig. 1). In total, 5 rats were partially resistant to acute food restriction (ABA_V: *n* = 1, ABA_O: *n* = 3, ABA_P: *n* = 1, Table 3) and did not reach their target weight over the course of the whole experiment. Nevertheless, their data were included in all analyses because they remained in the acute ABA phase until finalization. The differences in the time to reach the target weight did not reach statistical significance (*p* = 0.45). Starting from the second day after the initiation of acute ABA, both the mean body weight (acute starvation: control: 153 g, ABA_V: 119 g, ABA_O: 123 g, ABA_P: 120 g; chronic starvation: control: 185 g, ABA_V: 106 g, ABA_O: 111 g, ABA_P: 110 g; Fig. 2a) and mean food intake (acute starvation: control: 19.29 g, ABA_V: 5.93 g ABA_O: 5.63 g, ABA_P: 6.23 g; chronic starvation: control: 20.34 g, ABA_V 9.13 g, ABA_O: 7.97 g, ABA_P: 8.58 g; Fig. 2b) in all ABA groups were significantly different from those in the control group (*p* ≤ 0.001). Over the course of the acute ABA phase, the ABA_V animals had a significantly greater RWA compared to the control animals (*p* = 0.0173, Fig. 2d), while the RWA of the ABA_O and ABA_P animals was not significantly greater than that of the control group (acute starvation: control: 197.62%, ABA_V: 362.58%, ABA_O: 263.49%, ABA_P: 277.44%; chronic starvation: control: 281.87%, ABA_V 307.76%, ABA_O: 200.53%, ABA_P: 250.95%, Fig. 2c and d). In addition, when comparing the ABA groups, we found that the ABA_V group had a significantly greater RWA during the complete starvation phase compared to the ABA_O group (#*p* = 0.018, Fig. 2c). Additionally, the ABA_P animals showed a tendency toward reduced RWA in comparison to the ABA_V group ($*p* = 0.076, Fig. 2c).

**Table 3:** Time until target weight and resistant animals.

Group	Days until target weight	Resistant animals
ABA_V	5.78 ± 2.63	1
ABA_O	6.83 ± 3.48	3
ABA_P	7.14 ± 2.71	1

### Reduction in GFAP-positive cells after chronic starvation

**Fig. 3:**
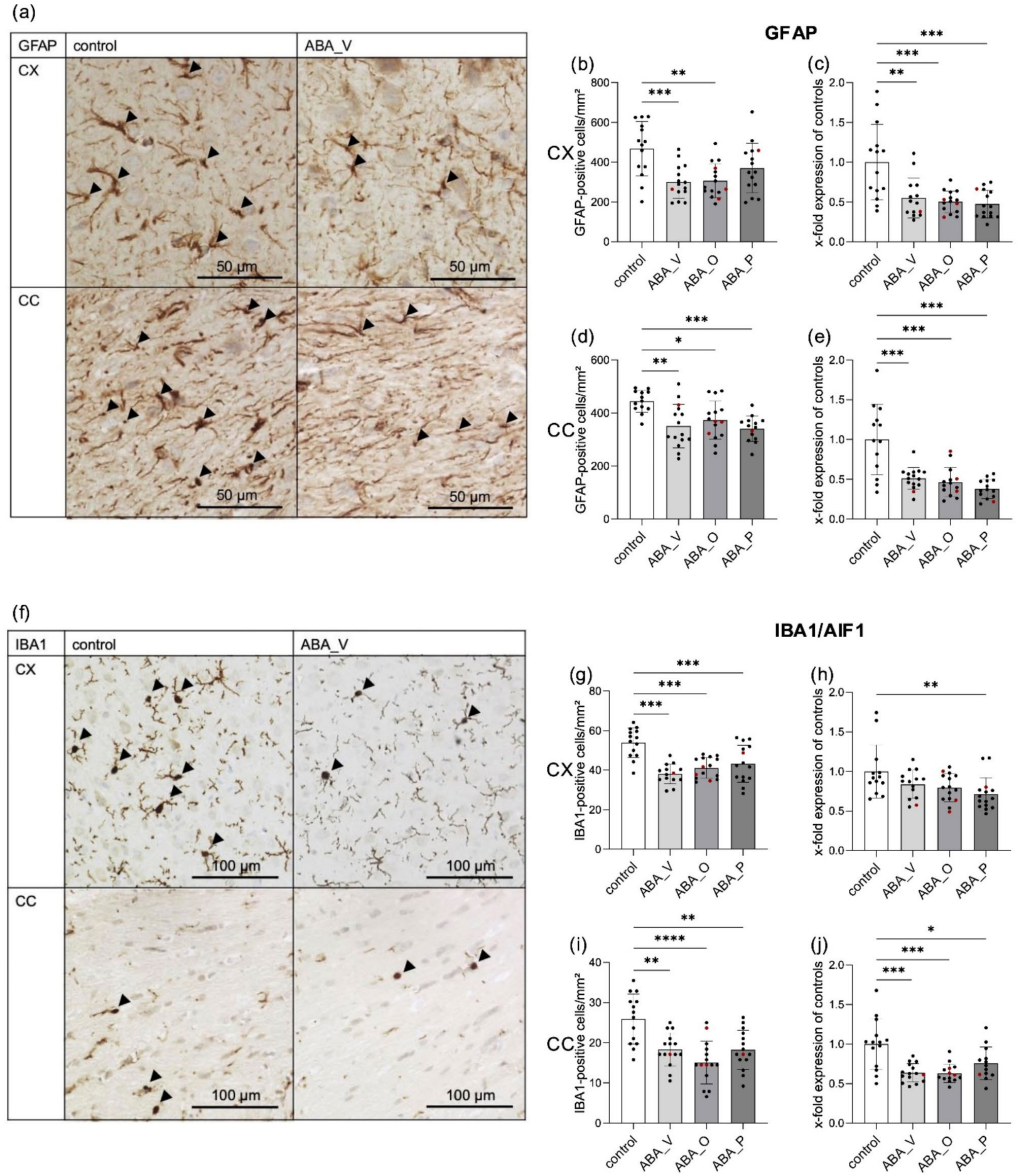
(**a**) Images of GFAP staining in the corpus callosum (CC) and cerebral cortex (CX) of control and ABA_V rats. GFAP-positive cells are indicated with arrowheads. (**b**) GFAP-positive cell count/mm^2^ (mean ± SD) in the CX, *n* = 59 (control *n* = 14, ABA_V: *n* = 15, ABA_O: *n* = 15, ABA_P: *n* = 15). (**c**) GFAP mRNA expression (mean ± SD) in the CX by qPCR, *n* = 58 (control: *n* = 14, ABA_V: *n* = 14, ABA_O: *n* = 15, ABA_P: *n* = 15). (**d**) GFAP-positive cell count/mm^2^ (mean ± SD) in the CC, *n* = 57 (control: *n* = 13, ABA_V: *n* = 15, ABA_O: *n* = 15, ABA_P: *n* = 14). (**e**) GFAP mRNA (mean ± SD) in CCs by qPCR, *n* = 56 (control: *n* = 13, ABA_V: *n* = 15, ABA_O: *n* = 14, ABA_P: *n* = 14). (**f**) Images of IBA1 staining in the corpus callosum (CC) and cerebral cortex (CX) of control and ABA_V rats. IBA1-positive cells are indicated with arrowheads. (**g**) IBA1-positive cell count/mm^2^ (mean ± SD) in the CX, *n* = 58 (control: *n* = 14, ABA_V: *n* = 14, ABA_O: *n* = 15, ABA_P: *n* = 15). (**h**) Quantification of AIF1 mRNA (mean ± SD) in the CX by qPCR, *n* = 57 (control: *n* = 13, ABA_V: *n* = 14, ABA_O: *n* = 15, ABA_P: *n* = 15). (**i**) IBA1-positive cell count/mm^2^ (mean ± SD) in the CC, *n* = 59 (control: *n* = 14, ABA_V: *n* = 15, ABA_O: *n* = 15, ABA_P: *n* = 15). (**j**) AIF1 mRNA (mean ± SD) in the CC by qPCR, *n* = 57 (control: *n* = 14, ABA_V: *n* = 15, ABA_O: *n* = 14, ABA_P: *n* = 14) *p ≤ 0.05, **p ≤ 0.01, ***p ≤ 0.001, one-way ANOVA with post hoc Bonferroni correction. *ABA_V* ABA animals with water interventions, *ABA_O* ABA animals with omega-3 FAs interventions, *ABA_P* ABA animals with probiotic interventions. Individual values from resistant animals are highlighted in red.

To determine the relative density of astrocytes in the CX and CC, the density was examined and compared to the control group. Representative images of GFAP-stained ROIs from control and ABA_V animals are shown in Fig. 3a. In cortical tissue, the number of stained GFAP-positive astrocytes was significantly lower in the ABA_V and ABA_O groups than in control animals, which were defined as 100% (ABA_V = 64%, *p* = 0.008; ABA_O = 66%, *p* = 0.001; Fig. 3b). Compared with relative cell density in control animals, the extent of astrocyte reduction in the CX in ABA_P was not significantly different (79%, Fig. 3b). At the mRNA level, all ABA-treated animals exhibited significantly lower *GFAP* expression in the CX than did the control animals (ABA_V: *p* = 0.0024; ABA_O: *p* = 0.0008; ABA_P: *p* = 0.001; Fig. 3c). In the CC, the number of stained GFAP-positive astrocytes in all ABA-treated animals was significantly lower (ABA_V = 79%, *p* = 0.0018; ABA_O = 84%, *p* = 0.0282; ABA_P = 77%, *p* = 0.0006; Fig. 3d). Similarly, at the gene expression level, all ABA-treated animals expressed significantly less *GFAP* than did the control group (ABA_V: *p* = 0.009; ABA_O: *p* ≤ 0.001; ABA_P: *p* ≤ 0.001; Fig. 3e).

### Reduction in IBA1-positive cells after chronic starvation

The relative microglial density in the CX and CC was compared to that in the control group. Exemplary images of IBA1-stained sections of control and ABA_V animals evaluated in this study are shown in Fig. 3f. In the CX, the number of IBA1-positive microglia in all ABA groups was significantly lower than that in the control group (ABA_V = 71%, *p* ≤ 0.001; ABA_O = 76%, *p* ≤ 0.001; ABA_P = 80%, *p* = 0.0009; Fig. 3g). This effect was not supported at the mRNA level for all groups. Compared with those in the control group, *AIF1* expression was significantly lower in the ABA_P intervention group (*p* = 0.0072; Fig. 3h); however, the expression of *AIF1* in the ABA_V and ABA_O groups was also decreased. The number of IBA1-positive microglia in the CC was significantly lower in all ABA groups than in the control group (ABA_V = 71%, *p* = 0.0013; ABA_O = 58%, *p* ≤ 0.001; ABA_P = 70%, *p* = 0.0011; Fig. 3i). This effect was also demonstrated at mRNA level (ABA_V: *p* = 0.0002; ABA_O: *p* = 0.0002; ABA_P: *p* = 0.046; Fig. 3j).

### Reduction in the number of OLIG-positive cells after chronic starvation

**Fig. 4:**
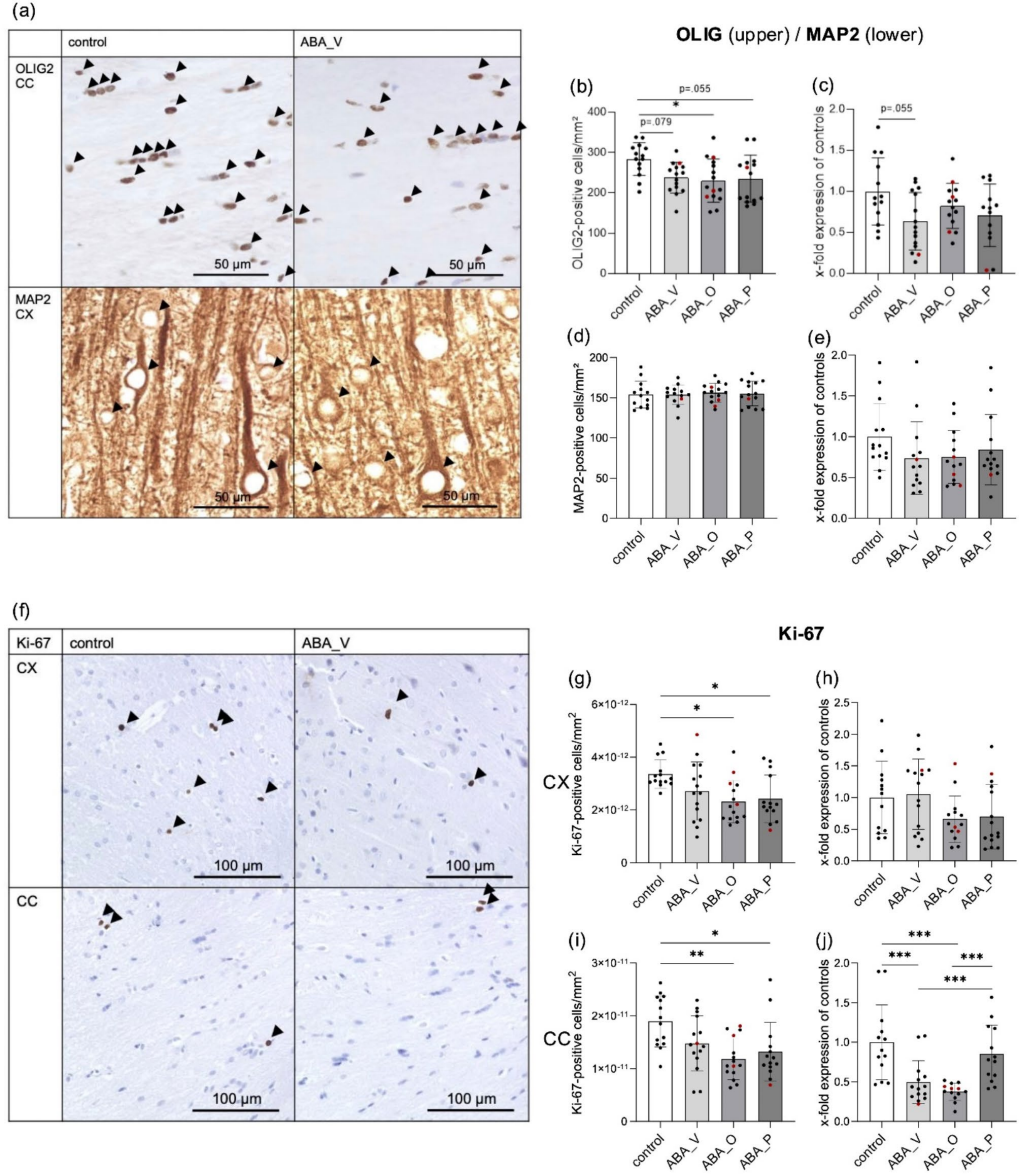
(**a**) Images of OLIG2 (CC) and MAP2 (CX) staining from control and ABA_V animals. OLIG2- or MAP2-positive cells are shown with arrowheads. (**b**) OLIG2-positive cell count/mm^2^ (mean ± SD), *n* = 59 (control: *n* = 14, ABA_V: *n* = 15, ABA_O: *n* = 15, ABA_P: *n* = 15). (**c**) OLIG1 mRNA (mean ± SD) by qPCR, *n* = 55 (control: *n* = 13, ABA_V: *n* = 15, ABA_O: *n* = 14, ABA_P: *n* = 13). (**d**) MAP2-positive cell count/mm^2^ (mean ± SD) *n* = 57 (control: *n* = 13, ABA_V: *n* = 14, ABA_O: *n* = 15, ABA_P: *n* = 15). (**e**) MAP2 mRNA (mean ± SD) by qPCR, *n* = 56 (control: *n* = 14, ABA_V: *n* = 14, ABA_O: *n* = 14, ABA_P = 14). (**f**) Images of Ki-67 staining in the corpus callosum (CC) and cerebral cortex (CX) of control and ABA_V animals. Ki-67-positive cells are shown with arrowheads. (**g**) Ki-67-positive cell count/mm^2^ (mean ± SD) in the CX, *n* = 58 (control: *n* = 14, ABA_V: *n* = 15, ABA_O: *n* = 15, ABA_P: *n* = 14). (**h**) Ki-67 mRNA (mean ± SD) in the CX by qPCR, *n* = 57 (control: *n* = 13, ABA_V: *n* = 15, ABA_O: *n* = 14, ABA_P: *n* = 15). (**i**) Ki-67-positive cell count/mm^2^ (mean ± SD) in the CC, *n* = 58 (control: *n* = 13, ABA_V: *n* = 14, ABA_O: *n* = 15, ABA_P: *n* = 14). (**j**) Ki-67 mRNA (mean ± SD) in the CC by qPCR, *n* = 53 (control: *n* = 13, ABA_V: *n* = 14, ABA_O: *n* = 13, ABA_P = 13). *p ≤ 0.05, **p ≤ 0.01, ***p ≤ 0.001; one-way ANOVA with post hoc Bonferroni correction. *ABA_V* ABA animals with water interventions, *ABA_O* ABA animals with omega FAs interventions, *ABA_P* ABA animals with probiotic interventions. Individual values from resistant animals are highlighted in red.

To observe differences in relative oligodendrocyte density in the CC, the density was examined by IHC and compared with the density in CC of the control group. Representative images of OLIG2 staining from control and ABA_V animals are shown in Fig. 4a. The number of stained oligodendrocytes was nominally reduced for all ABA-treated animals. This reduction was statistically significant for ABA_O. There was a decreasing trend for ABA_V and ABA_P groups (ABA_V = 84%, *p* = 0.0790; ABA_O = 81%, *p* = 0.0253; ABA_P = 83%, *p* = 0.0555; Fig. 4b). At the mRNA level, no differences between the groups were observed; however, there was a trend toward reduced *OLIG1* expression for ABA_V (ABA_V: *p* = 0.0552; Fig. 4c). In addition, we evaluated OLIG1/2 in the CX. The Olig2 IHC showed no significant differences between groups (ABA_V = 94%, ABA_O = 82%, ABA_P = 90%, supplementary Fig. 2a). At the mRNA level *OLIG1* expression was significantly lower in all ABA-treated animals (ABA_V: *p* = 0.0384; ABA_O: *p* = 0.0152; ABA_P: *p* ≤ 0.0001%, supplementary Fig. 2b). Interestingly, ABA_V and ABA_O had a higher *OLIG1* mRNA expression than ABA_P in the CX (*p* = 0.0094 and *p* = 0.0248 for the ABA_V and ABA_O groups, respectively; supplementary Fig. 2b). We also evaluated APC in the CX and CC in IHC and qPCR, complementary to the OLIG1/2-experiments. Here, no differences were observed (supplementary Fig. 3).

### No effect on MAP2-positive cells after chronic starvation

Changes in the number of MAP2 positive neurons between treatment groups and the control group were compared. Representative images of MAP2 staining from control and ABA_V animals are shown in Fig. 4a. For MAP2-stained neurons, there were no significant differences between the groups in terms of cellular density (ABA_V = 100%, ABA_O = 102%, ABA_P = 101%, Fig. 4d) or mRNA expression (Fig. 4e).

### Reduction in Ki-67-positive cells in supplemented ABA animals after chronic starvation

Assessment of proliferating cells in the CX and CC was done by comparing the relative expression of the experimental groups to the control group. Representative images of Ki-67-stained samples from control and ABA_V animals are shown in Fig. 4f. In the CX, the number of Ki-67-positive proliferating cells was nominally lower in all ABA-treated animals than in the controls, but this difference was significant only for ABA_O and ABA_P (ABA_V = 81%; ABA_O = 69%, *p* = 0.0126; ABA_P = 73%, *p* = 0.0356; Fig. 4g). At the mRNA level, *Ki-67* in the CX was only nominally but not significantly reduced for ABA_O or ABA_P (Fig. 4h). In the CC, the number of Ki-67-positive cells was lower in all ABA-treated animals than in the controls, but the difference was significant only for ABA_O and ABA_P (ABA_V = 78%; ABA_O = 62%, *p* = 0.0015; ABA_P = 70%, *p* = 0.0191; Fig. 4i). At the mRNA level, *Ki-67* expression was significantly lower in the ABA_V and ABA_O treatment groups than in the control group (ABA_V: *p* = 0.0006; ABA_O: *p* ≤ 0.001). Here, differences between the groups were observed: ABA_P expressed significantly more *Ki-67* mRNA than the ABA_V and ABA_O groups (*p* = 0.0092 and *p* = 0.0002 for the ABA_V and ABA_O groups, respectively; Fig. 4j).

## Discussion

Our study reveals new insights into glial cell dynamics in a chronic ABA model. The role of glial cells (astrocytes, microglia, oligodendrocytes, and ependymal cells) in the central nervous system (CNS) is to support, protect, and maintain optimal neuronal function. Consistent with previous research, our study showed a reduction in GFAP-positive astrocytes in the GM (CX) and WM (CC) caused by food deprivation in the ABA model^22,40^, which was also shown in the dehydration-induced anorexia model^39^. Aligning with previous research, there were no significant changes in neurons and only a limited reduction in oligodendrocytes^40^. Interestingly, the number of microglia in all three food-restricted groups was markedly decreased compared to the control group after chronic starvation.

Additionally, oral supplementation with omega-3 FAs influenced behavioral parameters, such as reduced hyperactivity. Probiotic supplementation showed lower effectiveness but in the same direction. Oral supplementation had no observable influence on brain cell reduction in ABA-treated animals in the present study.

### Glial cells in response to ABA

#### Microglia

Microglia, as immune cells, regulate inflammation, synaptic plasticity, and the formation of neural networks^45^, responding quickly to pathological insults^46^. Microglia are known to play a role in the pathogenesis of neurodegenerative and psychiatric diseases^47,48^. Additionally, in human postmortem studies, an increase in activated microglia in suicidal patients with depression was found^49^.

To put our findings in perspective, in a recent ABA model study by *Zimmermann* et al., no difference in microglial cell density was found in the CC and hypothalamus after acute starvation in mice^50^, potentially due to the shorter duration of starvation. In a study using a dehydration-induced anorexia rat model, an increase in microglial cell density in the hippocampus and prefrontal cortex was found after acute starvation^51^. However, after chronic starvation of mice, *Zimmermann* et al. reported a significant decrease in the number of microglia in the CC but not in the hypothalamus compared to control animals^50^. These findings align with our observations, suggesting a potential link between prolonged starvation and microglial depletion in specific brain regions.

Additionally, a study using the dehydration-induced anorexia model, elevated levels of the inflammatory markers were reported (TNF-α, IL-6, and IL-1β), suggesting the possibility of an environment conducive to neuroinflammation with suggested correlation with neurodegeneration^51^. For patients with AN, studies have investigated the general state of inflammation in patients with AN, showing elevated levels of the same proinflammatory cytokines (TNF-α, IL-6, and IL-1β)^52,53^. As neuroinflammation was observed in AN animal studies in several brain regions related to bodyweight and food intake control it can be suggested that neuroinflammation could play a role in the pathophysiology of AN^54^.

#### Oligodendrocytes, astrocytes and neurons

For oligodendrocytes, we found only a minor decrease, suggesting a slower response to stress caused by malnutrition compared to that of astrocytes. In a study on chronically starved ABA-treated mice, a decrease in OLIG2 cell density in the CC was observed, suggesting potential changes in myelin^50^. Our study confirms results of a previous study that investigated APC-positive cells in a chronic ABA model. No changes were found in CX or CC between ABA rats and controls in both studies^40^.

Interestingly, patients with AN showed diminished structural connectivity in the CC, where oligodendrocytes are the primary cell type. At weight normalization, this effect seemed to be reversible^55^.

In contrast to the described reduction of GFAP-positive cells after chronic starvation in rodents^22,40^, postmortem studies of terminally ill patients with AN have reported astrogliosis in specific brain regions (nucleus accumbens, ventral tegmental area)^56,57^.

Previously, we showed that the reduction in the number of astrocytes is reversible in an ABA refeeding model^22^. *Hurley* et al. reported a reduced density of GFAP-positive astrocytes in susceptible animals exposed to ABA compared to resistant animals after a 10-day recovery^58^. To further interpret our findings, assessing the potential reversibility of the reduction of other glial cells, especially microglia, in ABA refeeding models would be valuable.

Studies on postmortem terminally ill patients with AN reported neuronal deformation with cytoplasmic shrinkage in orbitofrontal cortex/anterior cingulate cortex^56,57^. In animal studies, the number of neurons seems to remain unaffected by malnutrition. This is consistent with the possibility of brain volume restitution upon weight recovery, as neurons show only very limited regrowth^40^.

Our findings suggest that not only reduction in the number of astrocytes, but also a reduction in the number of microglia might contribute to the diminished brain volume observed in the ABA model and in patients with AN. For patients with AN, a correlation between reduced brain volume and illness duration was found^18^. Furthermore, it has been shown that morphological and cellular brain changes could partially explain typical symptoms of AN; such as anxiety, rigidity, and learning impairments^16^. The reduction in brain volume was largely reversible after body weight restoration, especially in the GM, in patients with AN and in ABA-treated rats^19,20,22,59^.

#### Proliferating cells

Like astrocytes, microglia and the progenitor cells of oligodendrocytes can also proliferate. We observed a decrease in the number of proliferating cells after chronic starvation. This finding strengthens the hypothesis that cell reduction is likely due to decreased proliferation, possibly caused by a catabolic state of the cells, which do not have enough energy to build new cells^22,60^. A total reduction in the number of proliferating cells after chronic starvation might affect not only astrocytes, but also, as observed, microglia. Oral supplementation with omega-3 FAs and probiotics may indirectly support cell proliferation by reducing inflammation and enhancing nutrient absorption, while their capacity to cross the blood-brain barrier could modulate Ki-67 expression^61,62^. The regenerative potential of glial cells and the findings from ABA studies showing almost no differences in astrocyte numbers and gene expression in ABA rats after a refeeding period suggesting that glial cells, especially astrocyte, could be responsible this effect^22,33^.

### Supplementation studies

We found some indications that supplementation with omega-3 FAs (and, to a lesser degree, multistrain probiotics) influenced the phenotypes of the chronic ABA-treated animals: they performed less compulsory running and experienced a (non-significant) longer delay to reach the target weight.

Compared with control animals, non-supplemented ABA-treated animals also ran more during acute starvation, but omega-3 FAs or probiotic supplemented animals did not. In the acute ABA phase, we frequently observed that ABA-treated animals chose to run on their wheel instead of eating during feeding time, likely leading to even faster weight loss. It is possible that because of this increased physical activity, the non-supplemented ABA-treated animals also experienced more rapid weight loss than did those in the groups supplemented with omega-3 FAs or probiotics. This led to omega-3 FAs group having the longest time to reach the target weight, with probiotics featuring an intermediate phenotype (6.83 days). However, the differences did not reach significance.

Concerning the RWA, only non-supplemented ABA-treated rats showed a significantly greater RWA than did controls in the acute ABA phase. This effect was mirrored during the chronic starvation phase: Within the ABA group, animals supplemented with omega-3 FAs had lower RWA during the complete starvation phase compared to non-supplemented rats, with a non-significant trend toward reduced RWA also observed in animals receiving probiotics. During acute starvation, all ABA-treated animals remained active on a high level compared to controls. As the control animals also showed continuously increasing RWA, probably because they become bigger and stronger, we did not observe any significant difference between the groups during chronic starvation anymore. This is in line with findings from previous studies, where a relative reduction of the RWA increase was found after a few days of voluntary starvation^15,63^. In patients with AN, supplementation with omega-3 FAs may thus potentially help reduce the compulsion to move excessively and hyperactivity; therefore, it could even help with weight rehabilitation. This is the first study to investigate the effects of probiotics or omega-3 FAs in an ABA model, so comparative studies regarding AN are lacking. A systematic review and meta-analysis of patients with attention deficit hyperactivity disorder revealed a positive impact of supplementation with polyunsaturated fatty acids (PUFAs) on clinical symptoms (one of which is hyperactivity) and cognitive performance^64^. In a randomized controlled trial (RCT) patients with AN who received omega-3 FAs showed improvements in autonomic functioning^65^. Our findings in the ABA model point in the same direction and suggest a beneficial effect toward less hyperactivity by omega-3 FAs supplementation.

In contrast to our hypothesis, we did not find any effects on brain cell alterations due to interventions with omega-3 FAs or probiotics. This could be due to insufficient doses, a too short duration, or too small of an effect of the interventions.

The behavioral findings, however, suggest that targeting the inflammatory or metabolic pathways of the gut-brain axis might offer new therapeutic possibilities for AN treatment. While interventions were initiated during the acute starvation phase to assess early modulation effects, we acknowledge this design differs from clinical practice, where treatment typically starts together with weight restoration. Starvation effects observed in the vehicle group still allow relevant comparisons. The effects of PUFAs and probiotics on AN is currently being investigated in two RCTs (DRKS00017130 and DRKS00017726). These trials will further our understanding of whether nutritional supplements are suitable for improving microbiome composition, weight gain, and gastrointestinal discomfort and for reducing inflammatory processes and, therefore, whether they could be implemented in multimodal AN treatment^66,67^.

### Limitations

We provide significant insights into the development of chronic starvation. However, to analyze brain cell dynamics, an additional time point immediately after acute starvation would be of interest. The used cell counting procedure does not allow to draw conclusions about cell densities of the entire CNS, but only for the specific ROI. In addition, this study did not establish a causal link between glial cell reduction and brain volume loss. In the future, this could be addressed by fluorescent double-labeling with glia cell markers and proliferation markers or the bromodeoxyuridine (BrdU) assay which is used to determine proliferation rates in vivo^68^.

As this study is an animal model for a psychiatric illness, not all aspects (e.g., fear of gaining weight and body image disturbances) are transferable from animals to patients.

## Conclusion

This study confirmed a reduction in the number of astrocytes in the chronic ABA model and revealed a decrease in the number of microglia. We found that a reduced cell proliferation rate was a possible cause of glial cell depletion. Reduced glial cell density in response to chronic starvation was observed in both GM and WM, potentially leading to the brain volume reduction observed in both ABA-treated animals and patients with AN. Within the ABA groups, omega-3 FAs-supplemented animals showed lower running wheel activity during starvation than those receiving water, with a similar but non-significant trend in probiotic-receiving animals. This finding suggested that supplementation with omega-3-FAs might be beneficial for reducing hyperactivity in patients with AN, potentially leading to faster weight gain. We demonstrated that implementing an interventional study in a chronic ABA model is feasible, thereby providing new opportunities for systematic investigations on supplementation or medication when treating the state of chronic starvation. In conclusion, our findings contribute to our understanding of the pathogenesis associated with AN and could aid in the development of new treatment strategies.

## Electronic supplementary material

Below is the link to the electronic supplementary material.

Supplementary Material 1

Supplementary Material 2

Supplementary Material 3

Supplementary Material 4

## Data Availability

Data is available upon reasonable request to the corresponding author.
