# Supplementary material for: Chronic starvation induces microglial cell depletion in an activity-based anorexia model
Source: Sci Rep. 2025 Apr 23;15:14132. doi: 10.1038/s41598-025-98237-z (PMC12019532; doi:10.1038/s41598-025-98237-z)
Supplement: Supplementary file 1 — Supplementary Material 1 [file 41598_2025_98237_MOESM1_ESM.docx]

*Supplementary Fig. 1*

*Mean days until target weight (defined as -25% of baseline weight ± SD), n = 40 (ABA_V = 14, ABA_O = 12, ABA_P = 14). ABA_V = ABA animals with water interventions, ABA_O = ABA animals with omega-3 FAs interventions, ABA_P = ABA animals with probiotic interventions.*

*Supplementary Fig. 2*

*(****a****) Olig2-positive cell count/mm² (mean ± SD) in the CX, n = 59 (control: n = 14, ABA_V: n = 15, ABA_O: n = 15, ABA_P: n = 15). (****b****) Olig1 mRNA (mean ± SD) in the CX by qPCR, n = 57 (control: n = 13, ABA_V: n = 15, ABA_O: n = 15, ABA_P: n = 13). * p ≤ 0.05, ** p ≤ 0.01, **** p ≤ 0.0001; one-way ANOVA with post hoc Bonferroni correction. ABA_V = ABA animals with water interventions, ABA_O = ABA animals with omega-3 FAs interventions, ABA_P = ABA animals with probiotic interventions. Individual values from resistant animals are highlighted in red.*

*Supplementary Fig. 3*

*(****a****) APC cell count/mm² (mean ± SD) in the CX, n= 53 (control: n = 14, ABA_V: n = 9, ABA_O: n = 15, ABA_P: n = 15). (****b****) APC mRNA (mean ± SD) in the CX by qPCR, n = 55 (control: n = 13, ABA_V: n = 14, ABA_O: n = 13, ABA_P: n = 15). (****c****) APC cell count/mm² (mean ± SD) in the CC, n= 53 (control: n = 14, ABA_V: n = 9, ABA_O: n = 15, ABA_P: n = 15). (****d****) APC mRNA (mean ± SD) in the CC by qPCR, n = 57 (control: n = 14, ABA_V: n = 15, ABA_O: n = 14, ABA_P: n = 14). ABA_V = ABA animals with water interventions, ABA_O = ABA animals with omega-3 FAs interventions, ABA_P = ABA animals with probiotic interventions. Individual values from resistant animals are highlighted in red.*
