# Supplementary figures and images for: Chronic starvation induces microglial cell depletion in an activity-based anorexia model
Source: Sci Rep. 2025 Apr 23;15:14132. doi: 10.1038/s41598-025-98237-z (PMC12019532; doi:10.1038/s41598-025-98237-z)

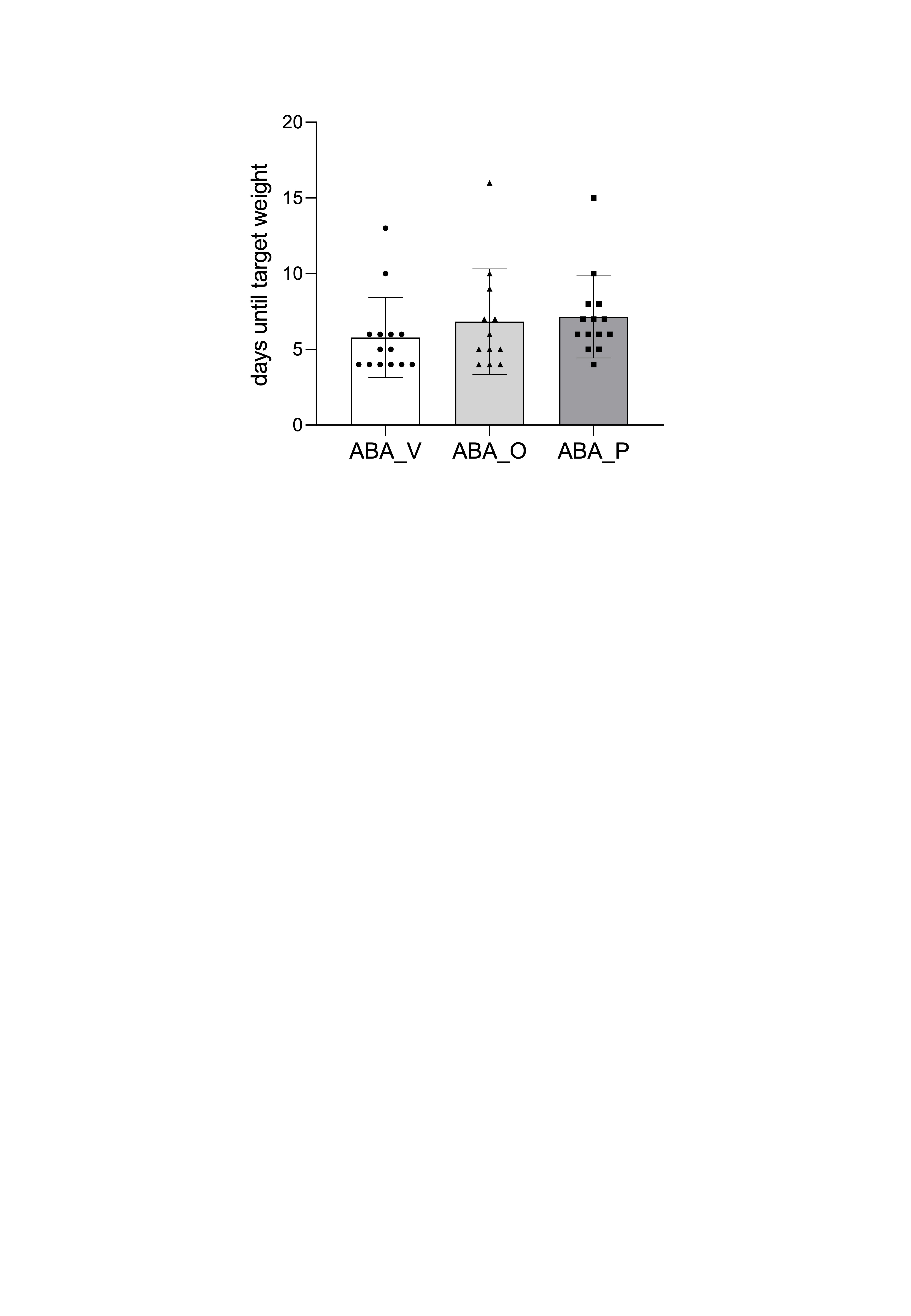

Supplement: Supplementary file 2 — Supplementary Material 2 [file 41598_2025_98237_MOESM2_ESM.jpg]

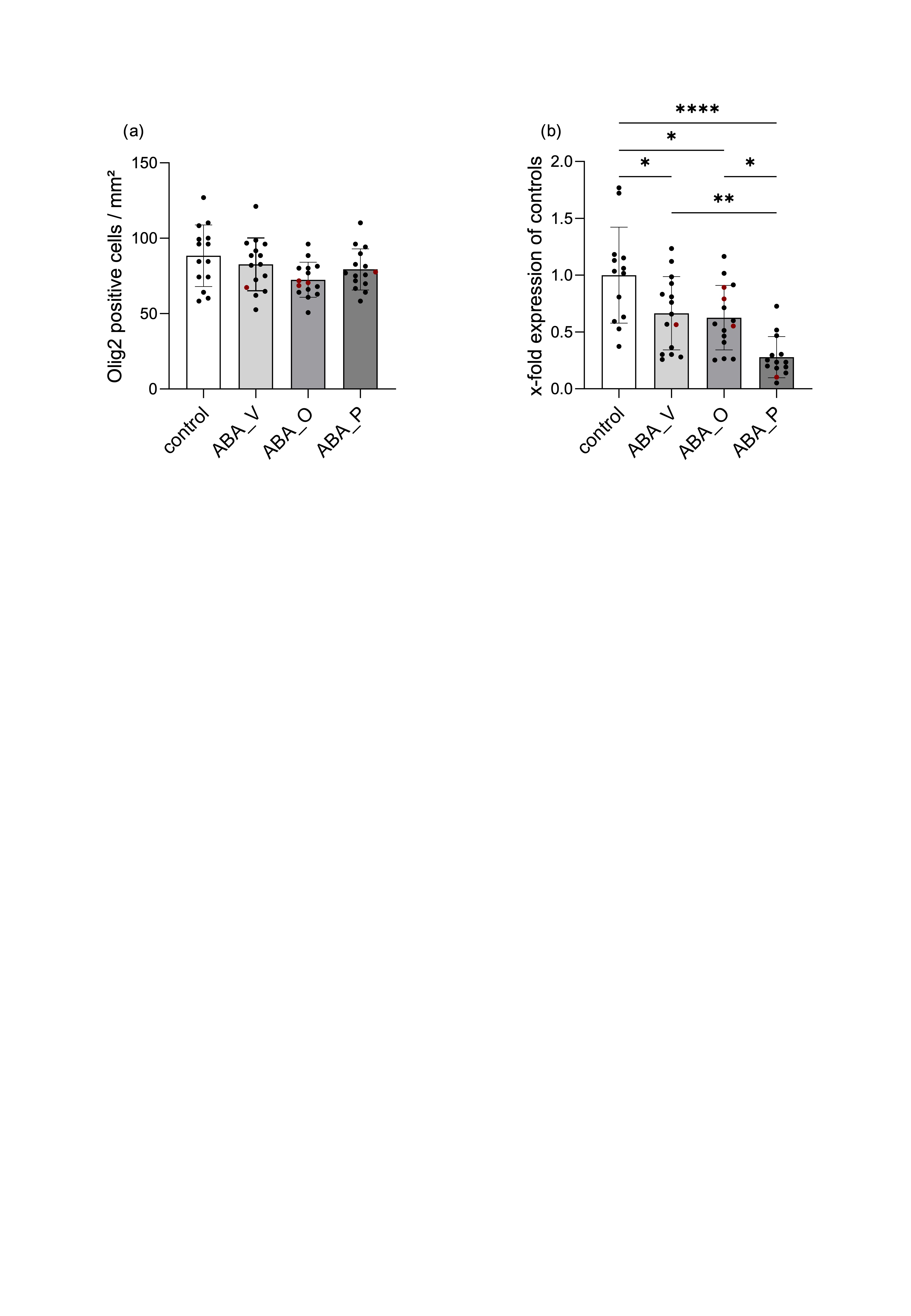

Supplement: Supplementary file 3 — Supplementary Material 3 [file 41598_2025_98237_MOESM3_ESM.jpg]

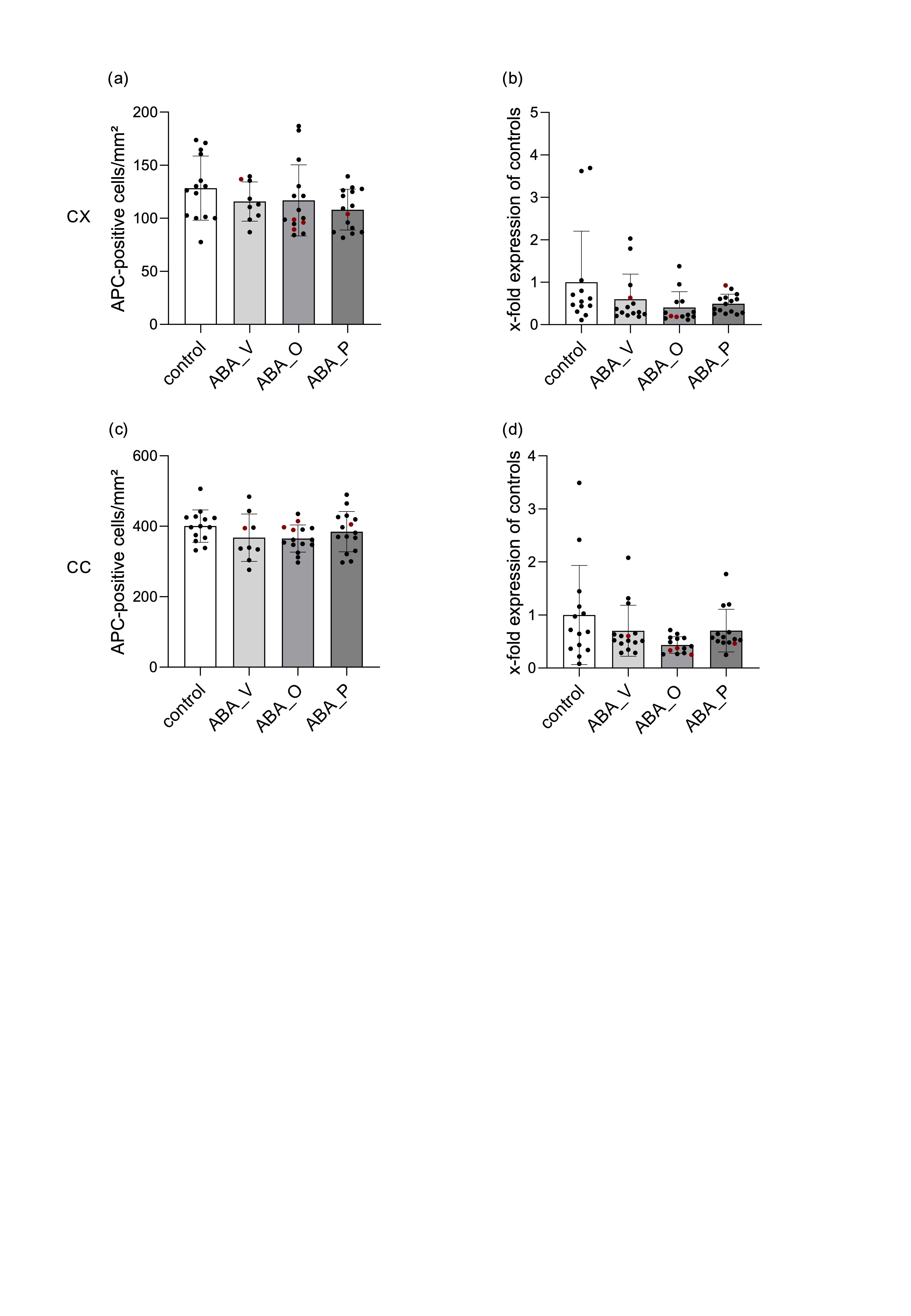

Supplement: Supplementary file 4 — Supplementary Material 4 [file 41598_2025_98237_MOESM4_ESM.jpg]
